# Physiological Adaptations to Hypoxic vs. Normoxic Training during Intermittent Living High

**DOI:** 10.3389/fphys.2017.00347

**Published:** 2017-05-31

**Authors:** Stefan De Smet, Paul van Herpt, Gommaar D'Hulst, Ruud Van Thienen, Marc Van Leemputte, Peter Hespel

**Affiliations:** ^1^Department of Kinesiology, Exercise Physiology Research Group, KU LeuvenLeuven, Belgium; ^2^Athletic Performance Center, Bakala Academy, KU LeuvenLeuven, Belgium

**Keywords:** altitude training, high-intensity interval training, muscle hemodynamics, pH regulation, EPO, hemoglobin mass

## Abstract

In the setting of “living high,” it is unclear whether high-intensity interval training (HIIT) should be performed “low” or “high” to stimulate muscular and performance adaptations. Therefore, 10 physically active males participated in a 5-week “live high-train low or high” program (TR), whilst eight subjects were not engaged in any altitude or training intervention (CON). Five days per week (~15.5 h per day), TR was exposed to normobaric hypoxia simulating progressively increasing altitude of ~2,000–3,250 m. Three times per week, TR performed HIIT, administered as unilateral knee-extension training, with one leg in normobaric hypoxia (~4,300 m; TR_HYP_) and with the other leg in normoxia (TR_NOR_). “Living high” elicited a consistent elevation in serum erythropoietin concentrations which adequately predicted the increase in hemoglobin mass (*r* = 0.78, *P* < 0.05; TR: +2.6%, *P* < 0.05; CON: −0.7%, *P* > 0.05). Muscle oxygenation during training was lower in TR_HYP_ vs. TR_NOR_ (*P* < 0.05). Muscle homogenate buffering capacity and pH-regulating protein abundance were similar between pretest and posttest. Oscillations in muscle blood volume during repeated sprints, as estimated by oscillations in NIRS-derived tHb, increased from pretest to posttest in TR_HYP_ (~80%, *P* < 0.01) but not in TR_NOR_ (~50%, *P* = 0.08). Muscle capillarity (~15%) as well as repeated-sprint ability (~8%) and 3-min maximal performance (~10–15%) increased similarly in both legs (*P* < 0.05). Maximal isometric strength increased in TR_HYP_ (~8%, *P* < 0.05) but not in TR_NOR_ (~4%, *P* > 0.05). In conclusion, muscular and performance adaptations were largely similar following normoxic vs. hypoxic HIIT. However, hypoxic HIIT stimulated adaptations in isometric strength and muscle perfusion during intermittent sprinting.

## Introduction

Since the 1968 Olympic Games in Mexico City (2,300 m) athletes have used altitude training to prepare for endurance exercise performances at sea-level. Extensive research has shown “living high” while “training low” to be the better approach in altitude training, because this strategy enables athletes to spend sufficient time in hypoxia to stimulate erythropoiesis whilst maintaining high mechanical work output and oxygen fluxes during training (Stray-Gundersen and Levine, 2008; Millet et al., 2010). Today, normobaric hypoxic installations are easily available. Thus athletes can continue living in their normal home environment, while “living high” in normobaric hypoxia, yet training at sea level. Indeed, hypobaric and normobaric hypoxic exposure recently were reported to be equally efficient in increasing total hemoglobin mass (Hbmass) (Hauser et al., 2016). The potential of “living high” to increase Hbmass (Gore et al., 2013; Garvican-Lewis et al., 2016b) and thereby raise maximal oxygen uptake rate (VO_2_max) (Robach and Lundby, 2012; Saunders et al., 2013) is now widely recognized. Nonetheless, considerable inter-individual variability exists in the responses to hypoxia (Chapman et al., 1998; Friedmann et al., 2005). Moreover, even within a given individual, Hbmass does not respond consistently to sequential altitude training exposures (McLean et al., 2013). Unfortunately, pertinent markers for individual altitude sensitivity are still lacking.

Hypoxia-induced stimulation of serum erythropoietin (sEPO) production drives the increase in Hbmass at altitude (Ge et al., 2002; Wenger and Hoogewijs, 2010; Jelkmann, 2011). The sEPO response to altitude is biphasic. sEPO as a rule peaks within 48 h, where after it gradually declines toward sea-level values over the following days and weeks (Abbrecht and Littell, 1972; Chapman et al., 1998, 2014; Garvican et al., 2012). This is probably at least partly due to increasing arterial oxygen content (Calbet et al., 2003; Lundby et al., 2004; Savourey et al., 2004), together with facilitated O_2_ extraction by EPO-producing cells in kidney and liver by virtue of elevated erythrocyte 2,3-diphosphoglycerate concentration (Klausen, 1998; Savourey et al., 2004). In addition, cells chronically exposed to hypoxia have been shown to lower their normoxic setpoint, hence blunting hypoxia signaling for the ongoing oxygen tension whilst allowing hypoxia-sensitive mechanisms to respond to new deviations in cellular oxygen availability (Khanna et al., 2006; Stiehl et al., 2006). It has been postulated that the magnitude of sEPO increment during the initial phase of altitude training can predict the eventual increment of Hbmass and even VO_2_ max (Chapman et al., 1998). However, this opinion was contradicted by later studies showing divergent sEPO and Hbmass responses in conjunction with high inter-individual variability (Friedmann et al., 2005; Clark et al., 2009; Garvican et al., 2012; Siebenmann et al., 2015). A strategy to maintain high sEPO levels during prolonged exposure to hypoxia might be to either gradually increase the degree of hypoxia, or discontinuously administer the hypoxic episodes to avoid desensitization.

Over the last two decades, interest in the beneficial effects of intermittent hypoxic training (IHT) has grown (Hoppeler et al., 2008; Faiss et al., 2013a). Indeed, a series of seminal studies reported greater upregulation of oxidative enzymes (Terrados et al., 1990; Melissa et al., 1997; Green et al., 1999) and mitochondrial biogenesis (Geiser et al., 2001; Vogt et al., 2001; Schmutz et al., 2010; Desplanches et al., 2014) in muscle following endurance training in hypoxia compared to similar training in normoxia. Nonetheless, published data with regard to the effects of IHT on sea-level exercise performance are equivocal (Hoppeler et al., 2008). Hypoxic training may upregulate myocellular oxidative potential, indeed, yet workload in endurance training sessions is reduced due to impaired oxidative energy provision (Wehrlin and Hallén, 2006). Therefore, attention has recently shifted to IHT in the form of sprint training, which allows for similar or even higher training intensities to be performed at altitude (Weyand et al., 1999; Hamlin et al., 2015). Furthermore, growing evidence indicates that hypoxic resistance training may stimulate muscle anabolism (Schoenfeld, 2013; Scott et al., 2014). Accordingly, heavy resistance training in hypoxia has recently been reported to induce greater strength gains than similar training in normoxia (Inness et al., 2016a).

The precise physiological mechanism underlying specific muscular adaptations to IHT remains to be elucidated. Training in hypoxia stimulates hypoxia-inducible factor-1α (HIF-1α) transcription (Vogt et al., 2001; Zoll et al., 2006; Faiss et al., 2013b; Brocherie et al., 2017b). In addition, acute hypoxia (10.7% F_i_O_2_), compared to normoxia, was recently found to stabilize HIF-1α protein in muscle both at rest and during submaximal exercise (Van Thienen et al., 2016). Repeated sprint training in hypoxia (RSH) has also been found to stimulate the transcription of monocarboxylate transporter 4 (MCT4) and carbonic anhydrase III (CA3) genes more than similar training in normoxia (Faiss et al., 2013b). Furthermore, a recent meta-analysis showed RSH, compared to similar training in normoxia, to induce greater improvements in sea-level repeated-sprint performance (Brocherie et al., 2017a). Yet sprint interval training in both normoxic and hypoxic conditions failed to increase muscular buffering capacity (De Smet et al., 2016). Recent observations also indicate that RSH may facilitate muscular perfusion during intermittent contractions (Faiss et al., 2013b, 2015; Montero and Lundby, 2017). Furthermore, there is evidence to indicate that training in hypoxia may stimulate angiogenesis in muscle tissue (Montero and Lundby, 2016).

The primary aim of the present study was to test the hypothesis that “living high” in conjunction with high-intensity interval training (HIIT) in hypoxia, compared to identical training in normoxia, enhances high-intensity exercise performance at sea level by stimulating beneficial muscular adaptations. In the current study, HIIT was administered in the form of unilateral knee-extensions in order to be able to compare hypoxic and normoxic training within the same individual during a single period of “living high.” Compared with whole body exercise, oxygen delivery to muscles during unilateral knee-extension exercise is markedly increased due to elevated capillary perfusion (Calbet and Lundby, 2012). Nonetheless, preliminary experiments in our laboratory showed that muscle oxygenation is significantly reduced by environmental hypoxia during intermittent knee-extension exercise, which makes the exercise model suitable for the purpose of this study. The secondary aim of this study was to assess the hematological and physiological responses to a discontinuous “living high” protocol during which altitude simulated by normobaric hypoxia was gradually increased.

## Materials and methods

### Participants

Following a medical screening, including a blood sample to assess iron status, 18 young healthy men (mean ± SD; 23.9 ± 3.0 y, 1.80 ± 0.07 m, 70.1 ± 6.6 kg) were recruited to participate in this study. Exclusion criteria upon admission were: exposure to altitude above 1,500 m in the 6 months prior to the study; intake of any medication or nutritional supplements during the 3 months prior the study; smoking; any kind of cardiopulmonary or musculoskeletal disorder. Subjects were recreational sports participants (4.8 ± 2.8 h per week, *P* = 0.76 between the experimental groups), yet none were engaged in training or sport at high competitive level. Subjects were instructed to maintain their habitual physical activities during the study period. Starting 1 week prior to the intervention, all subjects with serum ferritin concentrations (sFer) lower than 100 μg·L^−1^ received iron supplements delivering 105 mg elemental iron plus 500 mg Vitamin C per day (Ferograd^©^, Abbott S.p.A., Campoverde, Italy). The study was approved by the KU Leuven Biomedical Ethics Committee (B322201525619) and conducted in accordance with the Declaration of Helsinki. All subjects provided written informed consent after being fully informed about the content of the study and the risks associated with participation.

### Study design

**Figure 1:**
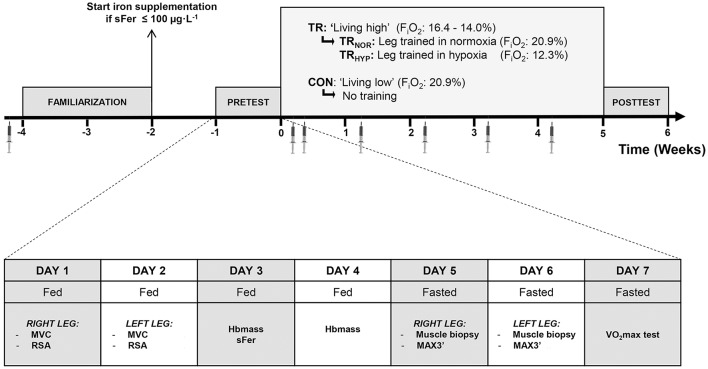
**Schematic presentation of the study protocol**. The study involved two experimental groups, o which one group was enrolled in a “live high” intervention using normobaric hypoxia (TR, *n* = 10), whilst the other group lived at sea level (CON, *n* = 8). In TR one leg was trained in 12.3% F_i_O_2_ (TR_HYP_, ~4,300 m), whilst the other leg was trained in 20.9% F_i_O_2_ (TR_NOR_). The week before (pretest) and the week after (posttest) the intervention period a series of measurements were performed. During the intervention period venous blood samples were taken on a weekly basis. MVC, maximal voluntary contraction; RSA, repeated-sprint ability; Hbmass, total blood hemoglobin mass; sFer, serum ferritin; MAX3′, 3-min maximal performance test (MAX3′); VO_2_max, maximal oxygen uptake rate.

The study involved two experimental groups, of which one was enrolled in a 5-week controlled “live high-train low or high” intervention using normobaric hypoxia (TR, *n* = 10), whilst the other group served as an untrained control group living low at sea level (CON, *n* = 8) (Figure 1). During this period, every Tuesday, Thursday, and Saturday, in TR but not in CON one leg was trained by knee-extension exercise at 12.3% F_i_O_2_ (~4,300 m; TR_HYP_), whilst the other leg was trained at sea level (TR_NOR_). Before (pretest) and at the end (posttest) of the intervention period a series of measurements was performed to assess physiological and exercise performance adaptations to “live high-train low or high.” Furthermore, muscle biopsies were taken from both the legs to evaluate muscular adaptations to TR_HYP_ vs. TR_NOR_. Between 1 and 3 weeks prior to the pretest, the subjects participated in 3 familiarization sessions with an interval of at least 2 days. These sessions served to habituate the subjects to the exercise tests to be performed in the pretest and the posttest. In the first familiarization session, subjects first performed a maximal voluntary contraction test and repeated-sprint ability test with the right leg. Following a 15-min rest period, similar testing was conducted with the left leg. In the second familiarization session, subjects first performed a 3-min maximal performance test with the right leg and subsequently with the left leg. The third familiarization session was similar to the second familiarization session, consisting of 3-min maximal performance testing of both the legs. Due to the nature of the intervention, subjects enrolled in TR were susceptible to experience an “expectancy-effect” which might lead to improved exercise performance in the posttest due to psychological factors (Beedie and Foad, 2009). In order to also induce an expectancy-effect in CON, all subjects received placebo supplements containing branched-chain amino acids (400 mg·day^−1^) and dry chicory powder (200 mg·day^−1^). The subjects were told that this novel “plant extract” was believed to have the potential to mimic altitude training effects on red blood cell mass.

### Normobaric hypoxia exposure-living high

**Figure 2:**
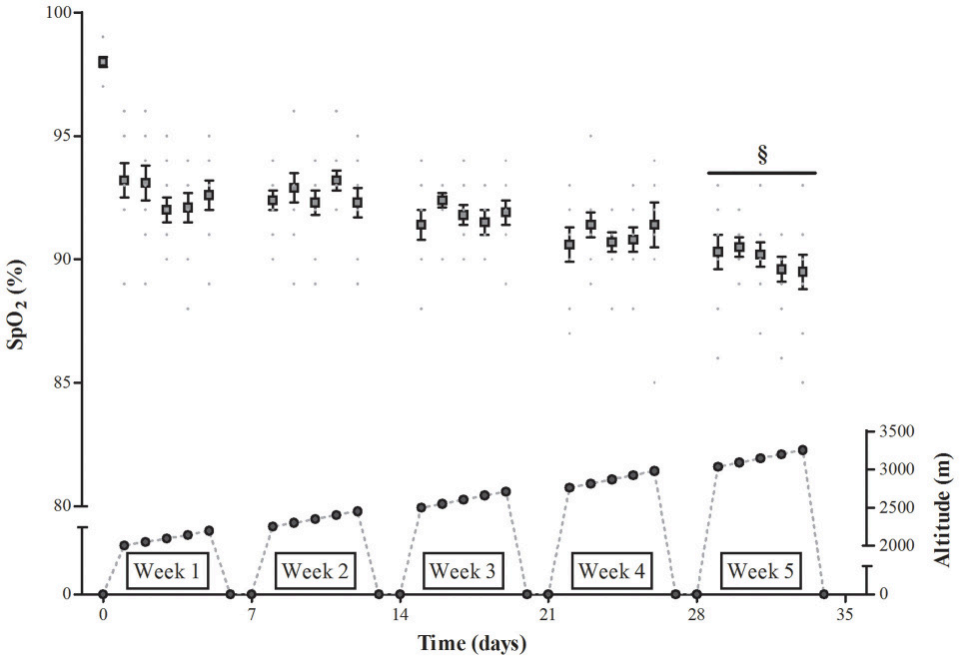
**Effect of incremental altitude on arterial oxygen saturation during “living high.”** %SpO_2_, Arterial oxygen saturation. §, *P* < 0.05 compared to week 1 at altitude.

The subjects in TR lived in a major hypoxic facility (232 m^2^; b-CAT, Tiel, The Netherlands), from Monday evening to Saturday morning. The facility (Bakala Academy, KU Leuven, Belgium) includes 5 large double bedrooms (~26 m^2^) and a major living/dining/recreational room (~70 m^2^) to spend non-sleeping time. On Saturdays and Sundays they lived in their normal home environment at sea level. On weekdays they spent on average ~15.5 h per day (range: 14.3–16.6 h) in the hypoxic facility, accumulating ~390 h in total (range: 378–395 h) over the 5-week intervention period, which in the conditions of the current study is equivalent to a hypoxic dose of 1,016 km·h (Garvican-Lewis et al., 2016b). The remaining time was spent outside the hypoxic facility at sea level. From day 1 to day 30 of the intervention period, F_i_O_2_ in the hypoxic facility was gradually decreased from 16.4% (~2,000 m altitude) to 14.0% (~3,250 m altitude) (see Figure 2). Every morning upon wake-up the subjects measured their arterial oxygen saturation (%SpO_2_) using a portable pulsoximeter (Nonin Medical, Onyx 9590, Plymouth MN, USA), filled in the Lake Louise Questionnaire (Roach et al., 1993) and registered the hours of effective sleep in a diary.

### Hypoxic vs. normoxic HIIT training

All training sessions were supervised by the investigators and consisted of unilateral knee extensions on a leg-extension apparatus (GLCE365, Body-Solid, Illinois, US). The leg-extension apparatus was adapted to limit knee-extension amplitude during training sessions from 95° to 175° knee angle. During each training session the subjects performed two sets of knee extensions with a 5-min passive recovery interval in between. Each set consisted of 4–6 series of 30 contractions at 20–25% of the 1-repetition-maximum (1RM), with 30 s of rest in between the series. The number of contraction series increased from four in sessions 1–5, to five in sessions 6–10, and eventually six in sessions 11–14. The relative load increased from 20% of 1RM in sessions 1–7 to 25% of 1RM in sessions 8–14. During each series the rate of muscle contraction and relaxation was paced by both auditory and visual feedback to obtain a 3-s duty cycle with 1s extension:1s relaxation:1s rest. 1RM was re-evaluated at the start of each next week to adjust training workloads. The subjects were randomly assigned to a group training with the dominant leg in hypoxia, whilst the contralateral leg was trained in normoxia, or vice versa. In each session the subjects thus trained one leg in the normobaric hypoxic facility (b-CAT, Tiel, The Netherlands) at 12.3% F_i_O_2_ (~4,300 m), whilst the contralateral leg was trained in an adjacent normoxic room at 20.9% F_i_O_2_. The subjects performed 3 training sessions per week, and the order of left vs. right leg training was alternated between the sessions. The oxygenation status of m. vastus lateralis was assessed by near-infrared spectroscopy (NIRS) during all training sessions in week 1 and week 5, as previously described (Van Thienen and Hespel, 2016). Immediately following each training session the subjects ingested 25 g of a standard whey protein concentrate mixture (Whey Shake, Sports 2 Health, Hofstade, Belgium) to stimulate muscular repair.

### Pretest and posttest protocol

The protocol for the pretest and the posttest was identical, and consisted of 7 experimental sessions (Figure 1). The experiments involved multiple unilateral knee-extension tests, an incremental VO_2_max test, biopsy sampling of m. vastus lateralis, and measurement of total hemoglobin mass. The order of the sessions was scheduled to avoid interference between the different measurements. From 48 h prior to the pretest and posttest the subjects were instructed to refrain from any strenuous activity other than the exercises prescribed by the study protocol. The last training session was scheduled 3 days prior to the posttest. All subjects consumed a standardized carbohydrate-rich dinner (~1,200 kCal; 65% carbohydrates, 15% fat, 20% protein) on the evening prior to each experimental day, and received a standardized breakfast (~960 kCal; 70% CHO, 10% Prot, 20% Fat) and lunch (~980 kCal; 64% CHO, 16% Prot, 20% Fat) on the next day. However, experimental sessions on day 5–7 were performed after an overnight fast. Furthermore, on the 7th day of the pretest, subjects spent the night in the hypoxic facility set at 20.9% F_i_O_2_. In the morning between 7 and 8 a. m. arterial oxygen saturation was measured upon wake-up (%SpO_2_, Nonin Medical, Onyx 9590, Plymouth MN, USA) and a blood sample was taken from an arm vein for assay of serum erythropoietin (sEPO) concentration (see below). These measurements were taken as the normoxic baseline. During the subsequent 5-week “living high” intervention %SpO_2_ was measured every morning upon wake-up, and blood samples for follow-up of sEPO changes were collected each Tuesday between 7 and 8 a.m. in identical conditions as in the pretest.

### Exercise testing procedures

#### Maximal voluntary contraction (MVC)

All knee-extension tests were performed separately for the left and the right leg. Maximal isometric contraction force was assessed on a self-constructed, motor-driven dynamometer as previously described (Vandenberghe et al., 1997). MVC was measured at a knee-angle of 135° extension (180° being fully extended). Subjects first warmed up by 3 sets of 30 submaximal dynamic contractions with 30-s rest intervals in between. Subsequently the subjects performed five 5-s maximal isometric knee-extensions, with a 1-min passive recovery interval in between. The highest mean torque (Nm) over a 2-s time frame was selected. The best performance out of five attempts was taken as MVC. The typical error for the MVC measurements was 6.2%, which is within the normal reliability range for MVC testing (Maffiuletti et al., 2007).

#### Repeated-sprint ability (RSA) test

The RSA test was performed on the same dynamometer following 10 min of passive rest after the MVC testing. Unilateral isokinetic knee-extensions from 95° to 175° knee angle were performed at a rate of 90°·s^−1^. Immediately after extension the leg was passively returned to the starting position (180°·s^−1^). The subjects were verbally encouraged to produce maximal force in each contraction. The RSA test consisted of 20 sets of 10 contractions. Each set was interspersed by a 15-s rest period (~1:1 work-to-rest ratio). The dynamic torque (Nm) productions were continuously digitized (250 Hz) by an on-line computer and both mean and peak (highest value out of 10 contractions) torque per set were automatically calculated. The typical errors for the overall mean and peak torque measurements were 3.5% and 3.7%, respectively.

#### 3-Min maximal performance test (MAX3′)

By analogy with the assessment of MVC and RSA, MAX3′ also was performed on the isokinetic dynamometer. The subjects first warmed up by 3 sets of 30 submaximal dynamic knee extensions (30% of MAX3′ in the familiarization sessions) with 30-s rest-intervals in between. Thereafter, subjects performed 140 maximal unilateral isokinetic contractions at a rate of 1.3 Hz (active extension and passive flexion movements at a rate of 90°·s^−1^ and 180°·s^−1^, respectively). Subjects were instructed to produce the highest possible total work output over the 3-min exercise bout. Initial target torque was taken from the familiarization sessions and real-time torque output was shown on a screen to assist the subjects in setting the optimal exercise intensity during the initial 30 contractions. Beyond 30 contractions the on-line feedback was stopped and only remaining time to finish was shown for the next 150 s. The typical error for the measurement of total work output in MAX3′ was 3.5%.

#### Incremental VO_2_max test

A maximal incremental exercise test was performed on a bicycle ergometer (Avantronic Cyclus II, Leipzig, Germany). Initial workload was set at 100 Watt and was increased by 20 W·min^−1^. Respiratory gas exchange was measured using a breath-by-breath open circuit spirometry system (Cortex Metalyzer IIIb, Leipzig, Germany). The highest average oxygen uptake over a 30-s time period was taken as VO_2_max. Heart rate was continuously monitored (Polar RS800CX, Kempele, Finland). One week after the posttest the subjects in TR performed an additional VO_2_max test to re-evaluate aerobic capacity following a 7-day return to sea level (~normoxia). The typical error for VO_2_max measurement was 3.4%, which corresponds with, or is favorable to literature data (Nordrehaug et al., 1991; Andersen, 1995; Fielding et al., 1997; Lourenco et al., 2011).

### Total hemoglobin mass assay

Hbmass was measured with a slightly modified version of the optimized CO-rebreathing procedure (Schmidt and Prommer, 2005), as previously described by Steiner and Wehrlin (2011). Briefly, a bolus CO of 1.0 mL per kg body mass was administered and rebreathed for 2 min. The percentage of carboxyhemoglobin was measured (ABL90 Flex, Radiometer, Copenhagen, Denmark) before (5 samples), and 6 and 8 min after administration of the CO bolus, by analysis of capillary blood samples (70 μL) taken from a preheated earlobe. The increase in carboxyhemoglobin was used to calculate the absolute Hbmass (g). A duplicate measurement of Hbmass was performed the next day. The typical error for the Hbmass measurements, calculated from the duplicate measures, was 1.8% which is in line with other work in the field (Gore et al., 2005).

### Blood sampling and analyses

During the medical screening and in the pretest and the posttest, blood (12 mL) was sampled from an arm vein into EDTA and serum separator tubes (Vacuette, Greiner Bio-One, Vilvoorde, Belgium) prepared for whole blood and serum analyses. Furthermore, additional blood samples were taken during the “living high” period (see above) for follow-up of sEPO. Samples were allowed to clot at room temperature before serum was separated by centrifugation and either immediately analyzed for serum ferritin concentrations (sFer) via immunoturbidimetric assay (Roche Modular P Analyser, Roche Diagnostics, Switzerland) or stored at −80°C for later analysis of sEPO. White blood cell count (DxH 800, Beckman-Coulter, Namen, Belgium) was used for control of possible infection-induced alterations in sFer. However, white blood cell count test results were consistently normal throughout the study in all subjects. sEPO concentration was assessed in duplicate using a commercially available sandwich ELISA kit (R&D Systems, Minneapolis, MN, USA). The intra-assay typical error was 4.6% which is within the margins reported by the manufacturer.

### Near-infrared spectroscopy

A Niro-200 NIRS instrument (Hamamatsu, Japan) was used to measure tissue oxygen index (TOI) and changes in oxyhemoglobin (ΔO_2_Hb), deoxyhemoglobin (ΔHHb) and total hemoglobin (ΔtHb) content in the vastus lateralis muscle during RSA testing in the pretest and the posttest, as well as during training sessions in the first and the last week of the intervention period. A NIRS probe was placed centrally on the belly of the vastus lateralis muscle after local shaving of the skin. An elastic non-compressive bandage was wrapped around the probe to prevent interference with external light and to avoid movement of the probe during testing. A surgical pen marked the margins of the probe on the skin in order to allow for identical repositioning of the probe in later testing. The NIRS signal was recorded at 2 Hz. Before analysis, all data points were processed with a Butterworth filter (4th order, cutoff frequency of 0.05 Hz) in a mathematical software program (Matlab R2011a, The Mathworks, Natick, MA, USA). This filter was part of a custom made Matlab script in which data frames were selected manually. The minimum value of every negative peak (i.e., at the end of the knee-extensions set for tHb) and the maximum value of every positive peak (i.e., at end of recovery intervals for tHb) during each of the 20 sets of the RSA test were selected to evaluate the magnitude of changes (Δ) in NIRS parameters. Typical error of the average ΔtHb throughout the RSA test during muscle contractions and recovery was 23.0 and 21.8%, respectively. The corresponding typical errors for ΔTOI were 19.7 and 20.6%, respectively. The corresponding typical errors for ΔHHb were 17.2 and 17.2%, respectively, and 17.0 and 17.1% for ΔO_2_Hb. During the training sessions in week 1 and week 5 mean values of TOI and tHb were calculated for each 5-s interval during the sets and for each 10-s interval during the recovery interval between the two sets. Due to the experimental design of the present study, typical errors of NIRS-derived parameters during training sessions could not be calculated.

### Muscle biopsies

Subjects reported to the laboratory between 6 and 11 a. m. after an overnight fast. Following a 30-min rest period in the supine position, a biopsy was taken from vastus lateralis muscle using a Bergström-type needle through a single 5-mm incision in the skin (2% xylocaine without epinephrine, 1 mL subcutaneously; Van Thienen et al., 2014). Muscle biopsies were taken by a medical doctor who accumulated extensive experience with the muscle biopsy procedure over years. Immediately following the biopsy, local pressure was applied until bleeding had completely stopped. The incision was then carefully sealed with adhesive strips (Steri-Strip, 3M Health Care, St. Paul, MN) and covered with a sterile plastic gauze (OpSite, Smith & Nephew, London, UK). Muscle samples were divided into two parts. One part was rapidly frozen in liquid nitrogen and stored at −80°C for biochemical analyses. The other part was frozen in isopentane cooled in liquid N_2_ and stored at −80°C for histochemical analyses.

### Analysis of muscle samples

#### Buffering capacity of homogenized muscle (βhm)

βhm was evaluated by the titration method as previously described (Edge et al., 2006). Briefly, 2–3 mg dry muscle (dm) was dissected from connective tissue and homogenized in a sodium fluoride containing buffer (33.3 μL 10 mM NaF·mg dm^−1^). The homogenates were warmed to 37.0°C in a warm water bath. Basal pH was measured with a microelectrode (MI-410, Microelectrodes, Bedford, NH, USA) connected to a pH meter (Lab 850, Schott Instruments GmbH, Mainz, Germany) and subsequently adjusted to pH > 7.1 with sodium hydroxide (0.02 M NaOH). Then, via titration of 2 μL hydrochloric acid (0.01 M HCl) pH was stepwise adjusted until pH reached values below 6.1. βhm was expressed as mmol H^+^·kg dm^−1^ required to decrease pH with a given unit. 23 samples were analyzed in duplicate. In accordance with previous reports (De Smet et al., 2016), the typical error of the βhm measurement was 5.4%.

#### Muscle fiber type composition and capillarization

Serial 7-μm-thick cryosections were cut with a cryostat at −20°C. Cryosections were blocked for 1 h in phosphate buffered saline (PBS) containing 1% bovine serum albumin (BSA). Slides were then incubated for 2 h in primary antibody against CD31 (DakoCytomation, Heverlee, Belgium) in a 1:500 dilution in 0.5% BSA in PBS. The cryosections were washed and incubated in a biotinylated rabbit anti-mouse IgG (H&L) antibody (DakoCytomation) (1:500 in 0.5% BSA in PBS) for 1 h at room temperature. Following a quick wash, slides were incubated overnight in primary antibodies against myosin heavy chain I (BA-F8, Developmental Studies Hybridoma Bank) and myosin heavy chain IIa (SC-71, Developmental Studies Hybridoma Bank) dissolved in PBS with 0.5% BSA (diluted 1:50 and 1:600, respectively). After washing, cryosections were incubated in appropriate conjugated secondary antibodies 488 goat anti-mouse IgG2 (Invitrogen) and Alexa Fluor 350 goat anti-mouse IgG1 (Invitrogen) in a 1:1000 dilution in 0.5% BSA in PBS. The slides were washed again and incubated in streptavidin (1:100 dilution in PBS) for 30 min, after which they were washed and incubated in cyanine (1:100 dilution in PBS) for 8 min. Cover slips were mounted with fluorescent mounting medium (DakoCytomation, Carpinteria, CA) after which type I muscle fibers, type II muscle fibers and capillaries were examined using a Nikon E1000 fluorescence microscope (Nikon, Boerhavedorp, Germany). Photos of the slides were analyzed with ImageJ software (version 1.41, National Institutes of Health, USA). Only fibers with adequate cross-sections showing no signs of distortion or folding were counted. On average (±SD) 181 ± 73 fibers were analyzed per biopsy.

#### Western blotting

Standard Western Blotting procedures have been described elsewhere (D'Hulst et al., 2013). Following homogenization and protein extraction of ~20 mg muscle tissue, 20–40 μg of proteins were loaded on sodium dodecyl sulfate polyacrylamide gels for electrophoretic separation. The proteins were then electro-transferred to a polyvinylidene difluoride membrane at 90 V for 100 min. Subsequently the membranes were blocked for 1 h in 0.1% Tween 20 Tris-buffered Saline (TBST) containing 5% fat free dry milk powder, after which they were emerged in one of the following primary antibodies for overnight incubation at 4°C: monocarboxylate 1 (MCT1; AB3538P, Millipore, Temecula, CA, USA), MCT4 (AB3316P, Millipore, Temecula, California), carbonic anhydrase III (CA3; AB135995, Abcam, Cambridge, UK) and Na^+^/H^+^ exchanger 1 (NHE1; AB126725, Abcam, Cambridge, UK). The membranes were then washed with TBST and incubated for 60 min at room temperature in the appropriate secondary antibody (horseradish peroxidase-conjugated anti-mouse or anti-rabbit; Sigma-Aldrich, Bornem, Belgium) for chemiluminescent detection of proteins. Membranes were scanned and quantified with Genesnap and Genetools software (Syngene, Cambridge, UK), respectively. Results are presented relative to a standard sample (pool) run on each blot and relative to GAPDH (cat no. 2118 14C10, Cell Signaling Technology, Danvers, MA) as a housekeeping gene which was unaffected by the experimental conditions.

### Statistical analysis

Statistical analysis was performed using IBM SPSS Statistics 23.0 (SPSS, Chicago, Illinois). In order to compare the effects of training in normoxia (TR_NOR_) vs. training in hypoxia (TR_HYP_) a 2-way repeated measures ANOVA (group x time) was performed, using TR_NOR_ and TR_HYP_ in the factor “group.” The effect of the “living high” intervention was evaluated using a 2-way repeated measures ANOVA (group x time) with CON and TR in the factor “group.” Because pretest values in CON were similar between the legs for all variables measured (*P* > 0.05 paired Students' *T*-test), values from both legs were averaged and considered as one data point. A 3-way repeated measures ANOVA was used to assess the effects of iron supplementation over time (pretest vs. posttest) as a within subject variable and both iron supplementation (iron vs. no iron) and group (CON vs. TR) as between subjects variables. Violations of sphericity were tested by Mauchly's Test of Sphericity. Whenever violations of sphericity occurred, Greenhouse-Geisser correction was applied. *Post-hoc* Students' *T*-tests with Bonferroni correction were used for multiple comparisons whenever ANOVA yielded a significant main or interaction effect. Changes in performance and hemodynamics during RSA in CON were evaluated by paired Student's *T*-test. 95% confidence intervals (CI) are reported for significant results. In addition, effect sizes are given as Cohen's d or partial eta squared ($\eta_{\text{p}}^{2}$). Pearson correlation coefficients were calculated to evaluate the relationship between variables. A probability level *P* < 0.05 was defined as statistically significant. All data are expressed as means ± standard error of mean (SEM) unless stated otherwise. Typical percentage errors of measurements were calculated based on changes from the pretest to the posttest in CON as previously described (Hopkins, 2000).

## Results

### Effect of “living high” on blood measurements and VO_2_max

#### %SpO_2_, sEPO and sleep quality

**Figure 3:**
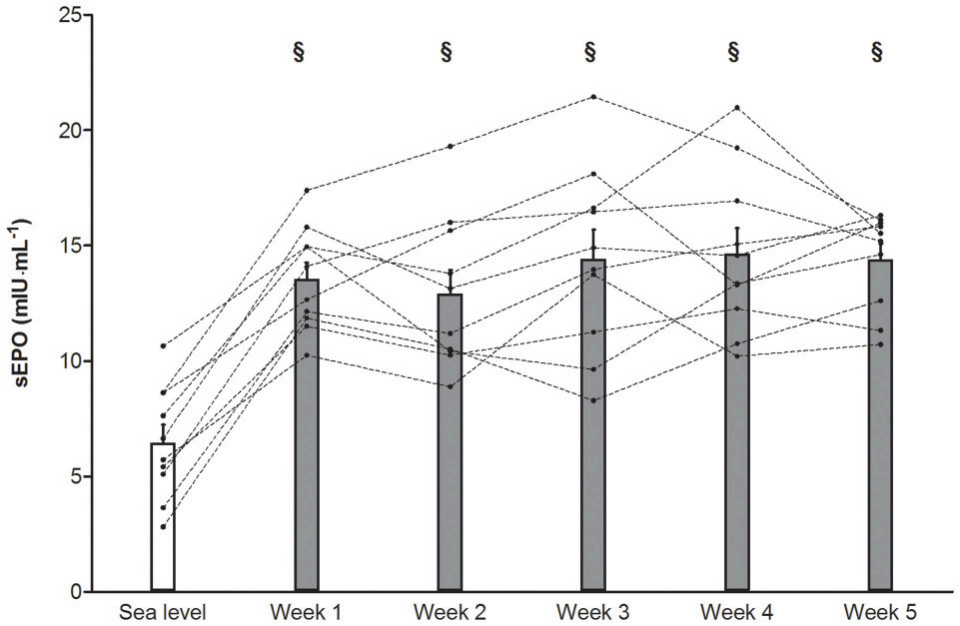
**Effect of “living high” on serum erythropoietin concentration**. Data are means ± SEM (*n* = 10) and individual data (dotted lines) for serum erythropoietin concentration (sEPO) in subjects living in normobaric hypoxia with simulated altitude gradually increasing from 2,000 m in week 1 to 3,250 m in week 5. See Methods for further details. §, *P* < 0.05 compared to sea level.

Compared to normoxia morning %SpO_2_'s in TR during the 5-weeks “living high” were consistently lower (*P* < 0.05, Figure 2). Furthermore, due to decreasing %F_i_O_2_ from week 1 to week 5, %SpO_2_ was slightly lower in week 5 (90.1 ± 0.2%, *P* < 0.05) than in week 1 (92.6 ± 0.3%). sEPO responses were highly variable between individuals (Figure 3). Compared to baseline, sEPO on average increased ~2-fold in week 1 (*P* < 0.05, CI: +4.4 to +9.8 mIU·mL^−1^) and this high level was maintained till week 5 (*P* < 0.05, CI: +4.9 to +11.0 mIU·mL^−1^). Subjective assessment of sleep quality was obtained from the Lake Louise Questionnaire, which rates sleep difficulty on a scale from 0 (slept as usual) to 3 (could not sleep at all). Sleep quality was similar between normoxia (0.1 ± 0.1) and hypoxia from week 1 (0.1 ± 0.0) to week 5 (0.3 ± 0.1). Sleep duration on average was 7.9 ± 0.1 h per night from the start to the end of the study. On average, scoring of acute mountain sickness by the Lake Louise Questionnaire in TR did not indicate incidence of AMS (total score of 3 or more) throughout the study. Nonetheless, compared to baseline values in normoxia (0.1 ± 0.1, range: 0–1), scores were significantly higher during hypoxia in week 5 (1.1 ± 0.2, range: 0–4, *P* < 0.05) with one subject testing positive for AMS.

#### Serum ferritin and hemoglobin mass

**Figure 4:**
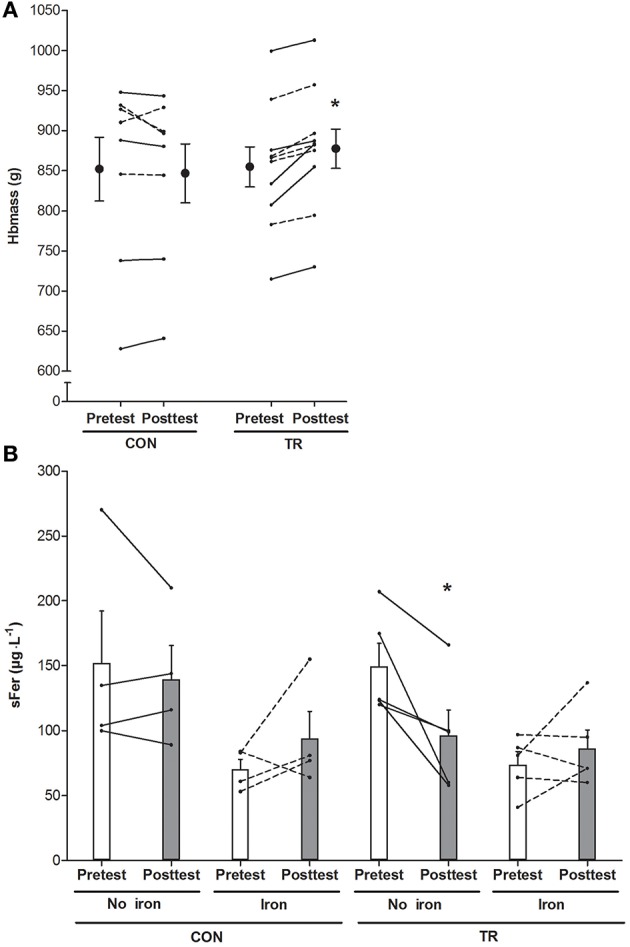
**Effect of living high on blood hemoglobin mass and serum ferritin concentration**. Data represent means ±SEM (n = 10) and individual values (lines) for hemoglobin mass (Hbmass, **A**) and serum ferritin concentrations (sFer, **B**) before (Pretest) and after (Posttest) living 5 weeks in normoxia (CON) or in normobaric hypoxia (TR) with simulated altitude gradually increasing from 2,000 to 3,250 m. Iron supplementation was provided in subjects with sFer below 100 μg·L^−1^ (dotted lines), but not in subjects with sFer above 100 μg·L^−1^ (solid lines). See Methods for further details. ^*^P < 0.05 compared to the pretest.

**Figure 5:**
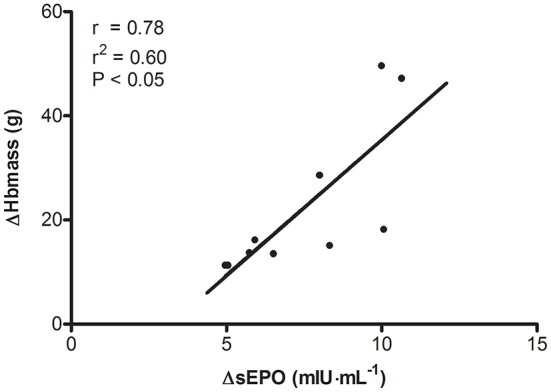
**Relationship between changes in serum erythropoietin concentration and changes in hemoglobin mass during “living high.”** ΔHbmass, change in total blood hemoglobin mass; ΔsEPO, mean increase in serum erythropoietin concentration during 5 weeks of normobaric hypoxia with simulated altitude gradually increasing from 2,000 to 3,250 m of simulated altitude.

Total Hbmass (*P* > 0.05, CI: −18 to +7 g, $\eta_{\text{p}}^{2}$ = 0.05) and sFer concentrations in CON were constant throughout the study (Figure 4). Conversely, in TR Hbmass on average increased by 2.6% from the pretest to the posttest (*P* < 0.05, CI: +11 to +33 g, $\eta_{\text{p}}^{2}$ = 0.54). In TR, in subjects not receiving supplementary iron because of high initial values (>100 μg·μL^−1^, *n* = 5), sFer decreased by ~30% from the pretest to the posttest (*P* < 0.05). Conversely, in subjects with low initial values who received iron supplementation (*n* = 5), sFer was constant at ~60–70 μg·μL^−1^ throughout the “living high” period. The average increase in sEPO (ΔsEPO, mIU·mL^−1^) over 5 weeks in TR was closely correlated with ΔHbmass (g) (*r* = 0.78, *P* < 0.05, Figure 5). In fact, correlations between ΔsEPO and ΔHbmass gradually increased from week 1 (*r* = 0.54, *P* = 0.09) to week 5 (*r* = 0.81, *P* < 0.05). No correlations were found between %SpO_2_ and either absolute sEPO concentration or ΔsEPO or ΔHbmass at any time.

#### VO_2_max

VO_2_max in CON was stable between the pretest (4.13 ± 0.21 L·min^−1^) and the posttest (4.11 ± 0.18 L·min^−1^). “Living high” in conjunction with knee-extension training did not significantly change VO_2_max either. VO_2_max in TR was 4.14 ± 0.11 L·min^−1^ in the pretest vs. 4.18 ± 0.15 L·min^−1^ in the posttest, and 4.24 ± 0.15 L·min^−1^ 2 weeks after return to normoxia following the posttest. In both the pretest (*r* = 0.85) and the posttest (*r* = 0.77) total Hbmass in CON and TR (*n* = 18) was closely correlated with VO_2_max (*P* < 0.05 for both). In contrast, in TR hypoxia-induced ΔHbmass did not correlate with ΔVO_2_max (*r* = 0.36, *P* > 0.05).

### Effects of hypoxic (TR_HYP_) vs. normoxic (TR_NOR_) training on muscular performance and oxygenation status

#### Measurements during knee-extension training

**Figure 6:**
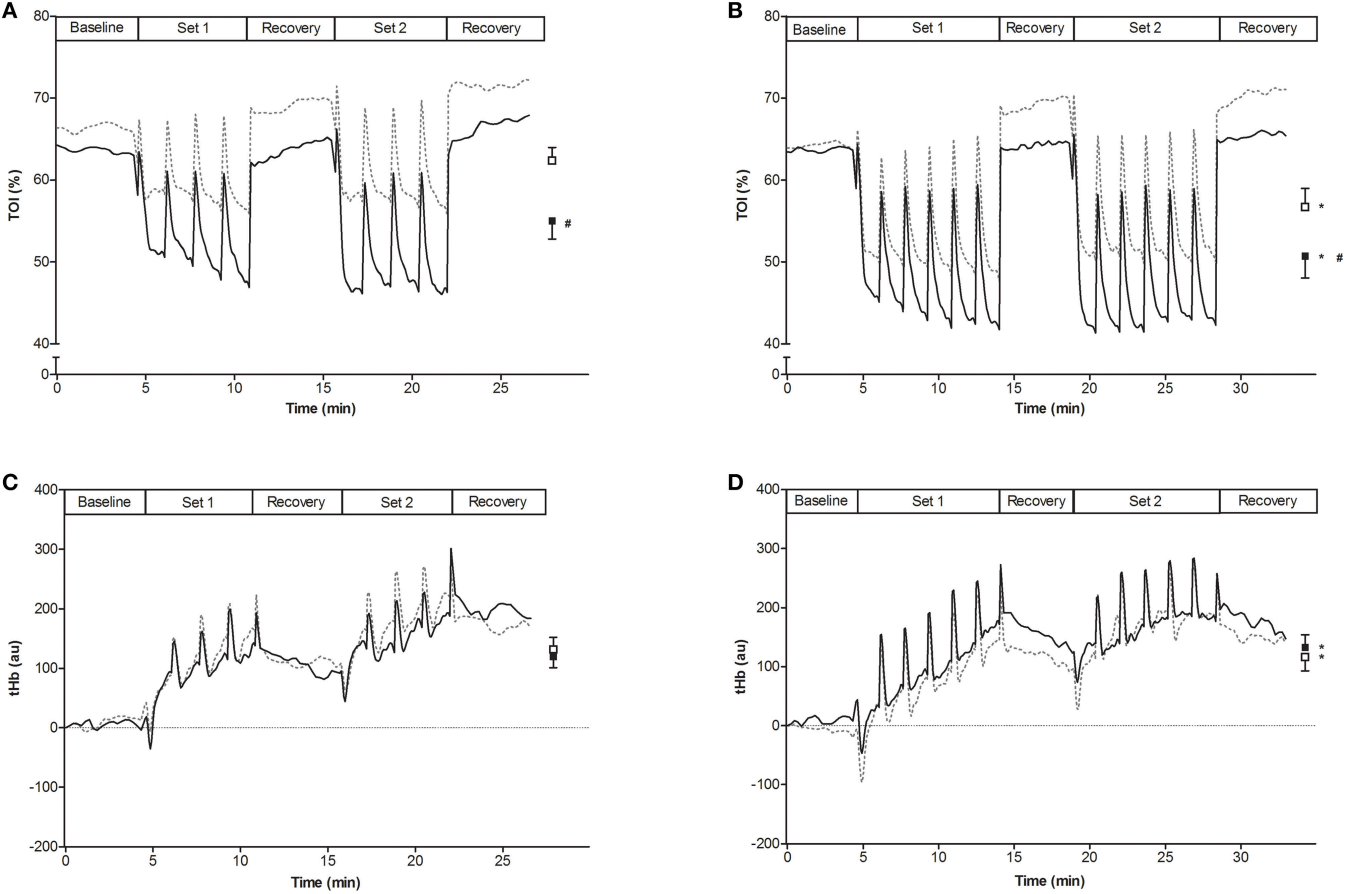
**Effect of knee-extension training in normoxia vs. hypoxia on muscle oxygenation status**. Curves represent mean values (*n* = 10) for tissue oxygenation index (TOI, **A,B**) and muscle total hemoglobin (tHb, **C,D**) measured by NIRS in subjects living for 5 weeks in normobaric hypoxia with simulated altitude gradually increasing from 2,000 to 3,250 m. Meanwhile one leg was trained in 12.3% F_i_O_2_ (TR_HYP_, ~4,300 m, full lines), whilst the other leg was trained in 20.9% F_i_O_2_ (TR_NOR_, dotted lines). The training sessions consisted of two times 4 series of 30 knee-extensions at 20% 1RM in week 1, increasing to two times 6 series of 30 knee-extensions at 25% of 1RM in week 5. Mean values ± SEM are given at the right side of each graph (TR_NOR_, □; TR_HYP_, ■). Values were measured in week 1 **(A,C)** and week 5 **(B,D)** of the training period. See Methods for further details. ^*^P < 0.05 compared to week 1; # *P* < 0.05 compared to training in normoxic conditions.

Training workloads on the knee-extension apparatus were adjusted weekly to the actual 1RM. 1RM significantly increased from the first (TR_NOR_, 40.7 ± 2.5; TR_HYP_, 42.4 ± 2.6 kg) to the last (TR_NOR_, 46.9 ± 2.4; TR_HYP_, 47.8 ± 2.4 kg) training week in both the legs(*P* < 0.05). Hence training workloads also were similar between TR_NOR_ and TR_HYP_ at any time of the study. Workloads in the initial training sessions on average were ~8.3 kg (range: 5.3–11.3 kg), increasing by ~25% to ~11.8 kg (range: 7.8–14.0 kg) in the final sessions. Maximal isometric contraction forces measured on a dynamometer significantly increased from the pretest to the posttest in TR_HYP_ (pretest, 230 ± 17 Nm; posttest 248 ± 15 Nm; *P* = 0.03, CI: +2 to +35 Nm, $\eta_{\text{p}}^{2}$ = 0.23) but not in TR_NOR_ (pretest, 236 ± 15 Nm; posttest 245 ± 14 Nm; *P* = 0.30, CI: −8 to +25 Nm, $\eta_{\text{p}}^{2}$ = 0.06), without significant difference between the groups. Corresponding values in CON were 208 ± 8 Nm and 210 ± 9 Nm in the pretest and the posttest, respectively (*P* > 0.05, CI: −14 to + 17 Nm, *d* = 0.08). To evaluate the effect of hypoxia on muscular oxygenation status during training, tissue oxygenation index (TOI) was measured by NIRS in m. vastus lateralis at the start and at the end of the training period. TOI during both the contraction and the recovery episodes consistently was ~10–15% lower in TR_HYP_ than in TR_NOR_ (Figure 6; week 1, *P* < 0.05, CI: −7.7 to −7.0 TOI percentage unit, $\eta_{\text{p}}^{2}$ = 0.13; week 5, *P* < 0.05, CI: −6.3 to −5.7 TOI percentage unit, $\eta_{\text{p}}^{2}$ = 0.12). Furthermore, TOI's were lower in week 5 than in week 1 in both the legs (TR_NOR_, *P* < 0.05, CI: −6.0 to −5.3 TOI percentage unit, $\eta_{\text{p}}^{2}$ = 0.09; TR_HYP_, *P* < 0.05, CI: −4.7 to −3.9 TOI percentage unit, $\eta_{\text{p}}^{2}$ = 0.06). The exercise-induced changes in tHb during training were similar between TR_NOR_ and TR_HYP_ at any time. However, in TR_NOR_ values were lower in week 5 than in week 1 (*P* < 0.05, CI: −21 to −10, $\eta_{\text{p}}^{2}$ = 0.003), whilst in TR_HYP_ values were higher in week 5 than in week 1 (*P* < 0.05, CI: +7 to +18, $\eta_{\text{p}}^{2}$ = 0.002).

#### Performance and hemodynamics in the RSA test

**Figure 7:**
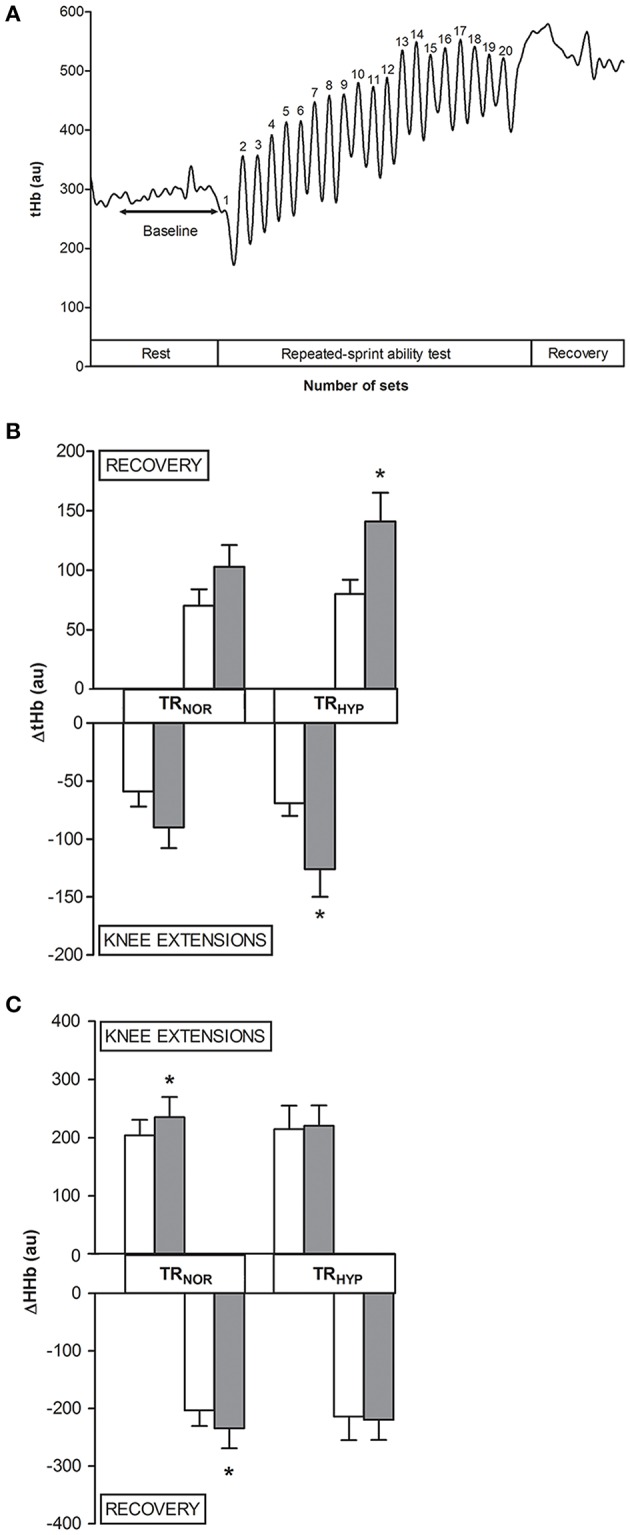
**Effect of knee-extension training in normoxia vs. hypoxia during “living high” on muscle total hemoglobin content during the repeated-sprint ability test. (A)** represents the typical signal of muscle total hemoglobin (tHb) measured by NIRS during the repeated-sprint ability test (*n* = 1), with tHb decreasing during consecutive knee extensions and increasing during recovery intervals. **(B,C)** represent means ± SEM for the change in tHb (ΔtHb) and HHb (ΔHHb), respectively, during the consecutive knee extensions and during the recovery intervals of the repeated-sprint ability test before (open bars) and after (solid bars) subjects living for 5 weeks in normobaric hypoxia with simulated altitude gradually increasing from 2,000 to 3,250 m. One leg was trained in 12.3% F_i_O_2_ (TR_HYP_, ~4,300 m), whilst the other leg was trained in 20.9% F_i_O_2_ (TR_NOR_). See Methods for further details. ^*^P < 0.05 compared to the pretest.

The RSA test consisted of 20 intermittent sets of 10 maximal unilateral knee extensions on an isokinetic dynamometer. Torque output in both TR_NOR_ and TR_HYP_ gradually decreased from set 1 to ~10 where after it was stable and there was no difference in percentage decrement score (Girard et al., 2011) between TR_NOR_ (pretest, 32 ± 3%; posttest, 34 ± 2%) and TR_HYP_ (pretest, 31 ± 3%; posttest, 32 ± 2%) at any time. Training increased mean torque production during the RSA test by ~8% in TR_NOR_ (pretest, 70 ± 3 Nm; posttest 76 ± 3 Nm, *P* < 0.05, CI: +3 to +9 Nm, $\eta_{\text{p}}^{2}$ = 0.47). Corresponding pretest and posttest values in TR_HYP_ were 74 ± 3 Nm and 80 ± 3 Nm (*P* < 0.05, CI: +3 to +9 Nm, $\eta_{\text{p}}^{2}$ = 0.51), respectively. By analogy with mean torques, training increased peak torque during the RSA test by ~8% from 85 ± 3 Nm to 92 ± 3 Nm in TR_NOR_ (*P* < 0.05, CI: +4 to +10 Nm, $\eta_{\text{p}}^{2}$ = 0.50), vs. from 90 ± 3 Nm to 96 ± 4 Nm in TR_HYP_ (*P* < 0.05, CI: +3 to + 10 Nm, $\eta_{\text{p}}^{2}$ = 0.49). In CON mean and peak torques values were constant at ~72 Nm (*P* > 0.05, CI: −4 to +2 Nm, *d* = 0.25) and ~87 Nm (*P* > 0.05, CI: −5 to +3 Nm, *d* = 0.18), respectively. During the RSA test tHb decreased during each series of contractions, where after it increased during the recovery intervals (Figure 7). ΔtHb's for both contraction and recovery phases were similar between TR_NOR_ and TR_HYP_ in the pretest, and in CON values were stable till the posttest (sprints, *P* > 0.05, CI: −15 to +34, *d* = 0.31; recovery, *P* > 0.05, CI: −16 to + 36, *d* = 0.33). However, training significantly increased ΔtHb's by ~80% during both the contraction and the rest episodes in TR_HYP_. Indeed, in TR_HYP_ ΔtHb during sprints increased from 69 ± 12 in the pretest to 126 ± 21 in the posttest (*P* > 0.05, CI: +22 to +93, $\eta_{\text{p}}^{2}$ = 0.39), whilst ΔtHb during recovery increased from 80 ± 13 in the pretest to 141 ± 22 in the posttest (*P* > 0.05, CI: +24 to +97, $\eta_{\text{p}}^{2}$ = 0.40). Conversely, in TR_NOR_ ΔtHb during sprints remained constant from the pretest (59 ± 12) to the posttest (90 ± 21, *P* = 0.08, CI: −4 to +67, $\eta_{\text{p}}^{2}$ = 0.16). Accordingly, also ΔtHb during recovery remained stable from the pretest (70 ± 13) to the posttest (103 ± 22, *P* = 0.08, CI: −5 to +69, η^2^p = 0.16) in TR_NOR_. Still ΔtHb values in the posttest were not significantly different between the two experimental conditions (*P* = 0.15). Furthermore, ΔHHb's were similar between TR_NOR_ and TR_HYP_ in both the pretest and the posttest, and in CON values were stable from the pretest to the posttest (sprints, *P* > 0.05, CI: −47 to +38, *d* = 0.09; recovery, *P* > 0.05, CI: −48 to +37, *d* = 0.10). However, ΔHHb's for both the contraction and rest intervals increased from the pretest to the posttest in TR_NOR_ (+~15%) but not in TR_HYP_ (+~3%). Indeed, in TR_NOR_ ΔHHb increased during sprints (pretest, 204 ± 34; posttest, 235 ± 35; *P* < 0.05, CI: +1 to +61, $\eta_{\text{p}}^{2}$ = 0.21) as well as during recovery (pretest, 203 ± 35; posttest, 235 ± 35; *P* < 0.05, CI: +1 to +61, $\eta_{\text{p}}^{2}$ = 0.21). In TR_HYP_, however, ΔHHb remained stable from the pretest to the posttest during both sprints (pretest, 215 ± 34; posttest, 220 ± 35; *P* > 0.05, CI: −24 to +36, $\eta_{\text{p}}^{2}$ = 0.01) and recovery (pretest, 214 ± 35; posttest, 220 ± 35; *P* > 0.05, CI: −25 to + 35, $\eta_{\text{p}}^{2}$ = 0.01). ΔO_2_Hb also increased from the pretest to the posttest in TR_NOR_ (+~27%, *P* < 0.05) and TR_HYP_ (+~24%, *P* < 0.05), whilst it was stable in CON. ΔTOI was unaffected by training and was similar between TR_NOR_ and TR_HYP_ at any time (*P* > 0.05, data not shown).

#### Performance in MAX3′

Training improved MAX3′ performance by about 10–15% in both the legs. Mean power output values in the pretest were 40 ± 2 and 40 ± 3 Nm for TR_NOR_ and TR_HYP_, respectively, increasing to 44 ± 2 and 46 ± 4 Nm in the posttest (*P* < 0.05 for both groups; TR_NOR_, CI: +2 to +6 Nm, $\eta_{\text{p}}^{2}$ = 0.45; TR_HYP_, CI: +3 to +8 Nm, $\eta_{\text{p}}^{2}$ = 0.59). Corresponding values in CON were 40 ± 3 and 42 ± 3 Nm (*P* > 0.05, CI: −1 to +4 Nm, *d* = 0.45).

### Effects of hypoxic (TR_HYP_) vs. normoxic (TR_NOR_) training on muscular adaptations

#### Muscle fiber type composition and capillarity (Table 1)

The relative number of type I and type II fibers was similar between the pretest and the posttest in both TR_NOR_ and TR_HYP_ (*P* > 0.05), and no overall training effect was found (*P* > 0.05). Type I and type II fiber cross-sectional areas (CSA) were constant from the pretest to the posttest in both TR_NOR_ and TR_HYP_ fibers. Capillary contacts per type I fibers increased similarly in TR_NOR_ (*P* < 0.05, CI: +0.3 to +1.2, $\eta_{\text{p}}^{2}$ = 0.37) and TR_HYP_ (*P* < 0.05, CI: +0.2 to +1.1, $\eta_{\text{p}}^{2}$ = 0.32). Accordingly, also capillary contacts per type II fibers increased similarly in both experimental conditions (TR_NOR_, *P* < 0.05, CI: +0.4 to +1.3, $\eta_{\text{p}}^{2}$ = 0.44; TR_HYP_, *P* < 0.05, CI: +0.1 to +1.0, $\eta_{\text{p}}^{2}$ = 0.28).

**Table 1:** **Effect of knee-extension training in normoxia vs. hypoxia during “living high” on muscle fiber type composition and capillary contacts per fiber**.

	**TR**_**NOR**_		**TR**_**HYP**_	
	**Pretest**	**Posttest**	**Pretest**	**Posttest**
**RELATIVE FIBER NUMBER (%)**				
Type I	58 ± 3 (42–71)	54 ± 4 (36–71)	55 ± 5 (31–73)	57 ± 4 (34–72)
Type II	42 ± 3 (29–58)	46 ± 4 (29–64)	45 ± 5 (27–69)	43 ± 4 (28–66)
**RELATIVE FIBER CSA (%)**				
Type I	45 ± 1 (42–49)	46 ± 1 (42–49)	46 ± 1 (41–50)	46 ± 1 (41–56)
Type II	55 ± 1 (51–58)	54 ± 1 (51–58)	54 ± 1 (50–59)	54 ± 1 (44–59)
**FIBER CSA (**μ**m**^2^**)**				
Type I	4614 ± 298 (3526–5530)	4477 ± 276 (3460–5587)	4639 ± 371 (3495–5995)	4162 ± 288 (3119–5185)
Type II	5452 ± 239 (4638–6154)	5300 ± 423 (4036–7092)	5610 ± 446 (3824–6873)	5276 ± 541 (3732–6874)
**CAPILLARY CONTACTS**				
Type I	5.0 ± 0.3 (4.1–6.6)	5.7 ± 0.4^*^ (4.4–8.2)	5.2 ± 0.3 (3.7–6.9)	5.8 ± 0.4^*^ (4.4–8.6)
Type II	4.8 ± 0.3 (3.9–7.0)	5.6 ± 0.4^*^ (4.3–8.0)	4.9 ± 0.3 (3.4–6.0)	5.5 ± 0.3^*^ (4.4–7.5)

*Data are mean ± SEM, and range in parentheses, before (Pretest) and after (Posttest) 5 weeks training in normobaric normoxia (TR_NOR_, n = 10) or hypoxia (TR_HYP_, F_i_O_2_: 12.3%, n = 10) while subjects lived in normobaric hypoxia with simulated altitudes gradually increasing from 2,000 m to 3,250 m. See Methods for further details*.

* *P < 0.05 compared to the pretest*.

#### Muscle pH regulation

Total buffer capacity measured in muscle homogenates (βhm) was similar between TR_NOR_ and TR_HYP_ in the pretest (164 ± 4 mmol H^+^·kg dm^−1^·pH^−1^ in both legs) and was unchanged in the posttest (TR_NOR_, 164 ± 3 mmol H^+^·kg dm^−1^·pH^−1^; TR_HYP_, 167 ± 3 mmol H^+^·kg dm^−1^·pH^−1^). The relative abundance of MCT1, MCT4, CA3 and NHE1 in muscle was not significantly altered by training and was similar between TR_NOR_ and TR_HYP_ in both the pretest and the posttest.

## Discussion

In this study, young healthy volunteers were exposed to a 5-week intermittent “living high” protocol during which altitude exposure, administered in the form of normobaric hypoxia, was gradually increased from ~2,000 m (F_i_O_2_: 16.4%) to ~3,250 m (F_i_O_2_: 14.0%). Meanwhile, subjects performed high-intensity interval-training (HIIT) with one leg in hypoxia equivalent to ~4,300 m (TR_HYP_, F_i_O_2_: 12.3%), whilst the contralateral leg trained in normoxia (TR_NOR_). It was postulated that lower muscular oxygenation status during HIIT in hypoxia, via activation of hypoxia-sensing pathways, could induce unique muscular adaptations to eventually enhance exercise performance. The 5 weeks of “living high” elicited a consistent elevation in serum erythropoietin (sEPO) levels which adequately predicted the eventual increment in total hemoglobin mass (Hbmass). Furthermore, compared with TR_NOR_, training-induced adaptations in the amplitude of muscle blood volume changes during intermittent high-intensity muscle contractions and maximal isometric strength were more pronounced in TR_HYP_. Nonetheless, HIIT during “living high” in general produced similar enhancements in exercise performance regardless of whether the training was performed in normoxia or hypoxia.

The primary aim of the current study was to compare the effects of hypoxic vs. normoxic HIIT during “living high.” Training workload was identical between TR_NOR_ and TR_HYP_ at any time, indeed, and increased by ~25% from the start to the end of the training period. Nonetheless, compared to TR_NOR_, muscle oxygen desaturation during exercise bouts was consistently exaggerated in TR_HYP_ (see Figure 6). Although, the possibility of collateral training adaptations via hemodynamic forces and humoral factors can not completely be excluded (Padilla et al., 2011), such effects are limited to absent when exercise involves small muscle groups (Katz et al., 1997; Miyachi et al., 2001; McGowan et al., 2007). Still, training responses were largely similar between the two experimental conditions, except for the exercise-induced oscillations in muscle blood volume and the increase in isometric strength. Muscle tHb content, measured by NIRS, was used as a marker for vascular blood filling (Truijen et al., 2012; Ihsan et al., 2013; Choo et al., 2016) during the RSA test. Muscle tHb content alternatingly dropped during exercise intervals, conceivably due to rise in intramuscular pressure, and increased during rest episodes (see Figure 7). Interestingly, however, the amplitude of tHb oscillations increased from the pretest to the posttest in TR_HYP_ but not in TR_NOR_. Concomitantly, unlike TR_NOR_, ΔHHb during contractions did not increase from the pretest to the posttest in TR_HYP_. Taken together, these observations indicate that higher power productions in the posttest in TR_NOR_ resulted in higher fractional O_2_-extraction, whilst in TR_HYP_ O_2_-delivery conceivably was increased due to elevated perfusion, yet at constant fractional O_2_-extraction rate. These findings corroborate recent observations (Faiss et al., 2013b, 2015; Montero and Lundby, 2017) showing more pronounced increases in the amplitude of tHb shifts during intermittent whole body sprinting in normoxia following a period of repeated sprint-training in hypoxia vs. normoxia. A potential mechanism to explaining these greater amplitudes in ΔtHb's in TR_HYP_, indicating greater blood volume shifts, is higher degree of arteriolar dilation in conjunction with increased capillary volume. Hypoxic training has previously been postulated to stimulate capillary growth by increased nitric oxide production in conjunction with increased vasodilation and endothelial shear stress (Hudlicka and Brown, 2009; Casey and Joyner, 2012) as well as by elevated HIF-1-induced VEGF gene expression (Breen et al., 1996; Tang et al., 2004; Van Thienen et al., 2016). However, literature data in this regards are equivocal with some studies showing hypoxic training to stimulate capillary density (Desplanches et al., 1993; Geiser et al., 2001; Vogt et al., 2001; Desplanches et al., 2014; Kon et al., 2014), vs. others showing similar adaptation between normoxic and hypoxic training (Terrados et al., 1990; Desplanches et al., 1996; Melissa et al., 1997; Masuda et al., 2001; Messonnier et al., 2001). In the conditions of the current study HIIT raised the number of capillary contacts per fiber by ~15% in both type I and type II muscle fibers, and irrespective of hypoxia. Still, this measure does not exclude higher capillary volume due to higher capillary tortuosity following training (Montero and Lundby, 2016; Olfert et al., 2016).

It was also postulated that hypoxic training during “living high” might promote muscular buffering capacity, which is pivotal in anaerobic performance. Stimulation of the HIF-1 pathway during hypoxic training is a potential mechanism to upregulate the expression of pH-regulating proteins such as monocarboxylate transporters, Na^+^/H^+^ exchangers and carbonic anhydrases (Porporato et al., 2011). However, regardless of whether HIIT was performed in normoxia or hypoxia, training altered neither the expression of muscle membrane proteins MCT1, MCT4, or NHE1, nor the abundance of cytoplasmic protein CA3. Furthermore, *in vitro* measured muscular total buffering capacity was also unaffected by training, independent of whether training was done in normoxia or hypoxia. Our current observations combined with literature data thus clearly indicate that short-term HIIT in hypoxia, by analogy with HIIT in normoxia (Mannion et al., 1994; Pilegaard et al., 1999a; Harmer et al., 2000; Bishop et al., 2008; Iaia et al., 2008; Baguet et al., 2011; De Smet et al., 2016; McGinley and Bishop, 2016a,b), is ineffective to increasing myocellular buffering capacity during either “living high” or “living low.”

pH-regulating proteins are differentially expressed between muscle fiber types, i.e., higher MCT1 and CA3 in oxidative vs. glycolytic muscle fibers (Fremont et al., 1988; Gros and Dodgson, 1988; Pilegaard et al., 1999b) and higher MCT4 and NHE1 in glycolytic vs. oxidative fibers (Pilegaard et al., 1999b; Juel, 2000). Hence training-induced muscle fiber-type shifts *per se* could alter the MCT1:MCT4 ratio, as well as total buffer capacity in mixed muscle tissue. 5 weeks of whole-body sprint interval training was recently demonstrated to decrease the fraction of type IIx fibers, irrespective of ambient oxygen concentrations (De Smet et al., 2016). However, in the present study we did not differentiate between type IIa and type IIx fibers, and the proportion of type I vs. type II fibers, either expressed as fiber numbers or cross-sectional areas, was constant throughout the study. With regard to fiber cross-sectional areas, some studies have indicated that hypoxia may stimulate muscle anabolism during resistance training (Nishimura et al., 2010; Manimmanakorn et al., 2013), whereas others did not (Friedmann et al., 2003; Kon et al., 2014). However, in keeping with earlier findings (Friedmann et al., 2003), the low-load resistance training program used in the present study stimulated muscle fiber hypertrophy neither in normoxia nor in hypoxia. In fact, literature data (Friedmann et al., 2003) taken together with our current observations indicate that if an exercise training program does not stimulate muscle mass accretion in normoxia, it probably will also fail to stimulate muscle hypertrophy in hypoxia. Still, maximal isometric strength increased by ~8% in TR_HYP_ vs. a non-significant increase of ~4% in TR_NOR_. Accordingly, heavy resistance training in hypoxia has recently been reported to stimulate adaptations in maximal strength despite not increasing lean mass (Inness et al., 2016a).

Another primary aim of the current study was to evaluate the potential additive effects of “living high” and hypoxic HIIT in stimulating high-intensity exercise performance. It is well established that “living high” can enhance performance by increasing total hemoglobin mass and thereby O_2_-delivery to muscles during exercise (Gore et al., 2013). Low arterial PO_2_ during sustained hypoxic exposure increases red blood cell production via HIF-2-induced stimulation and proliferation of renal EPO-producing and oxygen-sensing cells (Wenger and Hoogewijs, 2010). It is therefore surprising to note that literature mostly shows poor correlations between increased sEPO levels monitored during “living high” and eventual changes in hematological measurements (Friedmann et al., 2005; Clark et al., 2009; Garvican et al., 2012; Siebenmann et al., 2015). It is commonly postulated that sEPO represents the ratio between EPO synthesis and EPO degradation, and thus only represents an indirect marker of the erythropoietic signaling response. In addition, instantaneously measured sEPO levels may not adequately reflect sustained EPO action during prolonged “living high.” For circulating EPO levels often gradually decrease during short-term hypoxic exposure (Abbrecht and Littell, 1972; Chapman et al., 1998, 2014; Garvican et al., 2012), probably due to desensitization of the oxygen-sensing mechanism in EPO-producing cells consequent to HIF-α-induced induction of its negative regulators prolyl hydroxylase domain proteins 2 and 3 (Khanna et al., 2006; Stiehl et al., 2006), decreased oxygen hemoglobin affinity (Klausen, 1998; Savourey et al., 2004), as well as increasing arterial oxygen content (Calbet et al., 2003; Lundby et al., 2004; Savourey et al., 2004). In fact, only one (Chapman et al., 1998) out of multiple studies (Clark et al., 2009; Friedmann et al., 2005; Garvican et al., 2012; Siebenmann et al., 2015) found increments in both absolute and relative plasma EPO to be associated with changes in total red cell volume during “living high.” “Living high” in the conditions of the current study increased total Hbmass by ~2.6% on average. Moreover, this increase was highly correlated with the average sEPO increase over the 5-week study intervention (*r* = 0.78, *P* < 0.05). In fact correlations between ΔsEPO and ΔHbmass gradually increased from the start (week 1, *r* = 0.54) to the end (week 5, *r* = 0.81) of the intervention period. This supports the rationale that maintaining high circulating EPO levels during prolonged “living high” is crucial to increasing Hbmass at altitude. The current hypoxic protocol consistently elevated sEPO concentrations from week 1 to week 5 in all subjects. This is probably at least partly due to gradually increasing hypoxia from 16.4 to 14.0% F_i_O_2_ (~2,000 to 3,250 m), which resulted in even lower arterial oxygen saturations in week 5 than in week 1 (see Figure 2). Furthermore, within each week, 5 days in hypoxia were alternated with 2 days in normoxia. Follow-up studies are needed to evaluate whether intermittent hypoxia may be more effective than continuous hypoxia to stimulating EPO production for a given hypoxic dose.

According to a recent meta-analysis, “living high” is expected to increase Hbmass by ~1.08% for every 100 h of altitude exposure above 2,100 m (Gore et al., 2013). This algorithm predicts a ~4% increment in Hbmass for 350–400 h at >2,100 m in the current protocol. Accordingly, hypoxic dose expressed as “kilometer hours” predicts an increase of ~3.4% in Hbmass (linear model, Garvican-Lewis et al., 2016b). However, Hbmass increased by no more than 2.6% (range: 1.3–5.9%) in the present study. Correction for withdrawal of ~10 g of hemoglobin in ~65 mL blood during the study yields a net 3.7% increase in Hbmass. Nonetheless, one other study reported a 6.7% rise in Hbmass for only ~230 h of intermittent normobaric hypoxia, similar to the current protocol, yet at a constant altitude of 3,000 m (Inness et al., 2016b). This could indicate that intermittent hypoxia may become more effective to increase Hbmass at higher degrees of hypoxia. However, in order to maintain high sleep quality, which is important during altitude training in athletes (Sargent et al., 2013; Halson, 2014), initial altitude was set at only 2,000 m, reaching 3,000 m only in week 5 of the intervention. As a result, the magnitude of ΔHbmass probably was also insufficient to inducing a measurable change in aerobic power, because ΔVO_2_max is expected to rise by no more than 0.6–0.7% for each 1% increment of Hbmass (Saunders et al., 2013).

There is some evidence to indicate that iron availability is a primary factor limiting altitude-induced erythropoiesis, and that iron supplementation during “living high” may stimulate erythropoiesis (Stray-Gundersen et al., 1992; Gassmann and Muckenthaler, 2015; Govus et al., 2015; Garvican-Lewis et al., 2016a). In the present study, none of the subjects were diagnosed to be iron-deficient based on clinical criteria. However, given that retrospective observations in athletes indicate that both individuals with low (sFer < 20 μg·L^−1^) and normal (20–200 μg·L^−1^) sFer levels may benefit from iron supplementation to increase Hbmass during “living high” (Govus et al., 2015; Garvican-Lewis et al., 2016a), we decided to administer iron supplements (105 mg elemental iron per day) to all subjects exhibiting sFer concentrations lower than 100 μg·L^−1^ at baseline. Supplementary iron intake clearly abrogated the decrease in sFer in the subjects “living high” (see Figure 4). However, this did not alter the response of Hbmass to “living high.”

In the current study, “living high” in conjunction with hypoxic HIIT was found to increase total Hbmass and to stimulate oscillations in muscular perfusion during intermittent muscle contractions. It is reasonable to postulate that these effects eventually should contribute to enhancing performance in high-intensity exercise by increasing O_2_-delivery to muscles as well as by facilitating washout of metabolic end-products during rest episodes interspersing exercise bouts. However, performance in the repeated sprint-ability test was enhanced by HIIT, irrespective of whether training was performed in either normoxia or hypoxia. In this regard, however, it is important to note that the unilateral knee-extension exercise model chosen to assess exercise performance in the context of the current study may explain why the documented beneficial physiological adaptations did not translate into improved performance. Rather than blood flow and oxygen delivery, neuromuscular factors are believed to play the primary role in repeated sprint performance (Girard et al., 2011), especially during repeated sprints with small muscle mass. Indeed, it is well established that muscle blood flow is many fold higher during exercise involving a small muscle mass compared to whole body exercise (Savard et al., 1987; Calbet and Lundby, 2012). Therefore, extrapolations from the present study from single-leg HIIT in hypoxia, to whole-body HIIT in hypoxia should be performed with caution. Studies investigating whole-body exercise have produced equivocal results, with two studies showing improved intermittent cycling (Faiss et al., 2013b) or double-poling (Faiss et al., 2015) sprint performance following a period of RSH, vs. one other study showing no such ergogenic effects on repeated cycling sprint performance (Montero and Lundby, 2017). Clearly, more studies are needed to define the conditions wherein hypoxic training could enhance high-intensity intermittent exercise performance. It is also clear from the current and other studies (see above) that elevation of muscular buffering capacity is not a mechanism by which hypoxic HIIT could enhance anaerobic exercise performance.

In conclusion, this study demonstrates single-leg HIIT in hypoxia to stimulate adaptations in muscular perfusion during single-leg intermittent high-intensity muscle contractions, and to stimulate the increase in isometric strength, though without further inducing greater muscular and performance adaptations compared to similar training in normoxia. In addition, this study also shows that intermittent exposure to progressively increasing normobaric hypoxia elicits a stable increase in serum erythropoietin which adequately predicts the eventual increase in Hbmass. Follow-up studies are warranted to evaluate whether progressively increasing intermittent hypoxia may be more effective than continuous hypoxia to stimulate the increase in Hbmass.

## Author contributions

The experiments were performed at the Exercise Physiology Research Group, KU Leuven, Leuven, Belgium and at Bakala Academy–Athletic Performance Center, KU Leuven, Leuven, Belgium. Conception and study design: SD and Pv. All authors contributed to acquisition, analysis and/or interpretation of data for the work, and revised and approved the final manuscript for important intellectual content.

### Conflict of interest statement

The authors declare that the research was conducted in the absence of any commercial or financial relationships that could be construed as a potential conflict of interest.
